# Changes in soil organic carbon and its influencing factors in the growth of *Pinus sylvestris* var. *mongolica* plantation in Horqin Sandy Land, Northeast China

**DOI:** 10.1038/s41598-019-52945-5

**Published:** 2019-11-11

**Authors:** Zeyong Lei, Dongwei Yu, Fengyan Zhou, Yansong Zhang, Deliang Yu, Yanping Zhou, Yangang Han

**Affiliations:** 1 College of Environment Science and Engineering, Liaoning Technical University, Fuxin, Liaoning province China; 2 Liaoning Institute of Sandy Land Management and Utilization, Fuxin, Liaoning China

**Keywords:** Forestry, Climate change, Forest ecology, Forestry

## Abstract

The change of soil organic carbon and its influencing factors after afforestation in sandy land should be taken into account. Here, the factors would be revealed which would influence the SOC dynamics to a depth of 100 cm during the development of Mongolian pine plantations in Horqin sandy land, northeast China. The chronosequence method was used to quantify the change of SOC in vertical distribution and influencing factors following conversion grassland to *Pinus sylvestris* var. *mongolica* forest in semi-arid sandy land, northeast China. Then the traditional statistical approaches were used to assessed the influence of the identified factors. Stand age played a major role in SOC dynamics. It took 38 years for SOC in 0–10 cm layer to recover to its initial level after afforestation, and 46 years for 10–20 cm layer. SOC accumulation increased with the age of Mongolian pine plantation. Over-mature forest fully embodied the advantage of SOC accumulation. In addition, the changes of SOC in 0–10 cm layer were also affected by TN, TP, TK and soil moisture, and those below 10 cm soil layers were related to the effects of TN, TP, TK, BD and CS.

## Introduction

Land use change is the main factor that drives changes in soil organic carbon (SOC) stocks and the global carbon cycle^1^. SOC in the form of soil organic matter plays an important role in the formation and conservation of soil structure, soil nutrient cycling and soil biodiversity. SOC is a natural resource for the sustainable development of human society and a key foundation for sustainable forestry development^2^.

Studies on SOC are usually conducted near the soil surface (0–20 cm or 0–30 cm) and deep (below 20 cm or 30 cm) layers. Plant residues are the most important source of SOC. The SOC near the soil surface is mainly derived from aboveground litter and fine roots distributed near the soil surface layer, whereas deep SOC is mainly derived from root exfoliation and exudates^3^. The distribution of SOC is largely heterogeneous because the soil properties^4^, site characteristics^5,6^, plant species^1,7^, environment^4,5,8–12^, land use^7,11,13–15^ and management^16,17^, the dynamic carbon processes differ and the responses to external environmental changes are distinct between the surface and deep soil. However, results regarding the effect of time since conversion on SOC stock are inconsistent^17–20^. In small areas with the same climate and soil type, the factors that affect SOC include land use and management, plant species, site characteristics (slope position, aspect), intrinsic soil properties (e.g., BD, soil texture), stand age^18,19,21^, and stand density^22–24^. For example, in plains in Australia with rainfall less than 500 mm, different land uses (grass or planting) have no significant impacts on SOC^12^, but changing land management practices has been demonstrated to affect deep SOC^17^ and SOC stratification^16^.

After the conversion of grassland to forest land, the root system of the forest is able to regulate the redistribution of deep soil water^25^ and provide C^9^. Furthermore, the active plant roots can promote the formation of soil aggregates and make the most of the root-derived C that was occluded in soil aggregates^26^, thereby promoting the accumulation of soil organic matter in degraded grasslands^3^. There are a large of sandy grassland in northern China^27^. To increase the primary productivity of the land, the land use mode (grassland into agricultural or forest land) has been changed. Pine trees have high yields and low afforestation costs^28^, and introduced pine plantations are promoted for their presumed capacity to provide a net sink of atmospheric C^29^. Accordingly, *Pinus sylvestris* var. *mongolica* was first introduced to Horqin Sandy Land in 1955 and achieved good benefits^30^. Although some scholars have studied the effects of the pine plantations on SOC during growth, the maximum stand age that has been studied was 30 years old, and research has been limited to the 0–10 cm surface layer^31^, and Chen *et al*.^32^ also studied only 0–30 cm soil layer and certain stand age, not the whole growth process. Guo *et al*.^33^ concluded that carbon stocks were reduced by approximately 20% when plantations were less than 40 years old. Meta-estimates calculated for the periods <30 yrs and >30 yrs since afforestation revealed a shift from initial loss to later gain of SOC^34^. Chapela *et al*.^29^ demonstrated that during the 12 years after pine trees were planted on grassland, SOC remained almost unchanged in the 0–10 cm soil layer but decreased by 30% and 44% in the 10–20 and 20–30 cm soil layers, respectively. Therefore, estimates of the changes of SOC during the whole growth process may be inaccurate when only stand ages less than 30 years and the 0–10 cm or 0–30 cm soil layer are considered. The earliest introduced *Pinus sylvestris* var. *mongolica* individuals in Horqin sandy land have been growing for 56 yrs and have entered a stage of slow growth. How has SOC changed over these 56 yrs, and what are the relationships of SOC with other soil properties? How do *Pinus sylvestris* var. *mongolica* forests affect SOC?

The hypothesis that the drivers of SOC vary with stand age after afforestation with the exotic species *Pinus sylvestris* var. *mongolica* in sandy land, namely that the factors influencing the amount of SOC vary with stand age and also differ from those influencing the SOC of topsoil and deep soil layer. The method of stepwise regression analysis which was assessed the importance of various possible explanatory factors on the change of SOC in a depth of 100 cm was be used to test this hypothesis. The data of SOC and other soil properties were derived from 20 sites, laboratory testing and calculation from Horqin sandy land, northeast China.

## Material and Methods

### Study area

**Figure 1:**
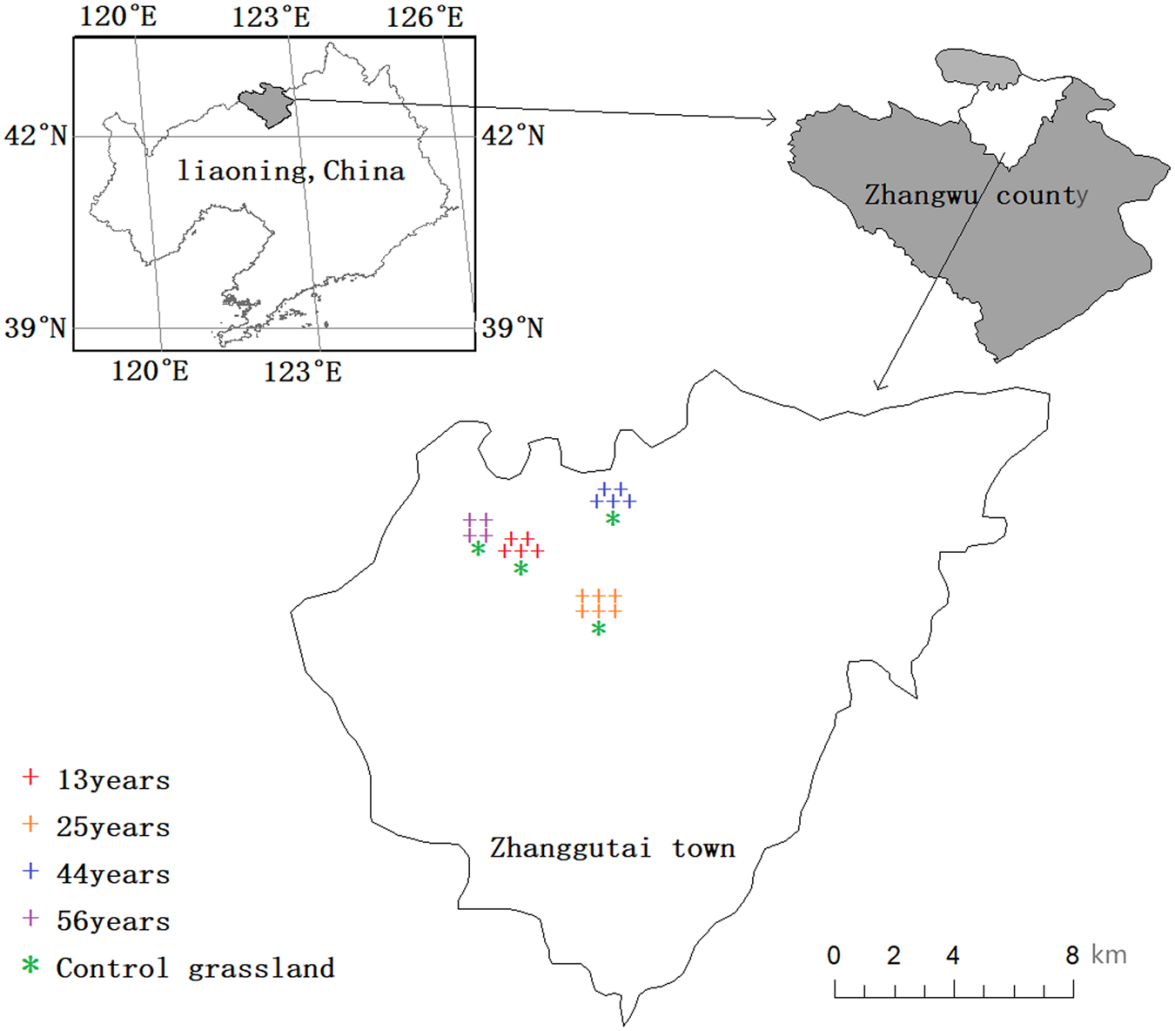
Location of the study area within Zhanggutai Town, Zhangwu County of Liaoning Province, China.

The research site is established in the southern part of the Horqin Sandy Land in Zhanggutai region of Liaoning Province, Northeast China (42°39′-42°43′N, 122°23′-122°33′E) with an average altitude of 225 m (Fig. 1).The area is a typical semi-arid sandy land with the annual average temperature of 4.6–6.3 °C and the average annual precipitation of 500 mm. The soil is eolian sandy type developed from sandy parent material through wind action. The annual average wind speed is 4.5 m·s^−1^, and winds can blow gusts of sand and dirt in spring. The area is a typical combination of grassland and agricultural land, representative plants include *Potentilla anserina*, *Cleistogenes chinensis*, *Armeniaca sibirica*, *Lespedeza daurica*, *Salix gordejevii* and *Agriophyllum squarrosum*^30^. Before afforestation, this area was a sandy grassland.

### Site selection

The survey was conducted in the above study area (Table 1). A paired forest land-grassland method was adopted, assuming that the SOC of the adjacent grassland represented the SOC at the initial stage of afforestation. Accordingly, the selection of the sample sites was based on the following criteria: (i) the forests should be planted on native grasslands and have soil properties similar to those in the adjacent grassland; (ii) plots should be on well-drained soils with no anoxic conditions influencing the decomposition rate of organic matter; (iii) sampled stands should, to the greatest extent possible, include young, middle-aged, mature and over-mature ages; and (iv) there should be 4–6 repetitions per stand age. In total, 20 suitable stands were identified and sampled and the site should have small slope and relatively flat slope. All the sampled stands were still in their first rotation after conversion from grassland. The growth stage was classified into four categories: young forest, with 13 yrs of afforestation, middle-aged forest, with 25 yrs; mature forest, with 44 yrs; and over-mature forest, with 56 yrs. There was a control grassland in each stand of forest age. After reviewing the afforestation archives, it was found that 2-year-old bare root seedlings of *Pinus sylvestris* var. *mongolica* were used during the original afforestation. Before afforestation, the herbs were removed with a diameter of approximately 20 cm. There was limited disturbance to the soil using this method. The soil in the study area was free of carbonate reactions (Xie 1982, Unpublished data). It means a lack of Ca^2+^ and Mg^2+^.

**Table 1:** Survey results for the sample plots, F stands for flat terrain site type, S.U. stands for slight undulate terrain.

Plot number	Stand age (yrs)	Stand density (plants per hectare)	Average tree height (m)	DBH (cm)	Site type
1	13	975	3.36	7.17	F
2	13	1625	3.66	7.27	F
3	13	1175	3.44	6.67	F
4	13	625	3.33	7.43	F
5	13	1425	3.82	7.36	F
6	25	1075	8.01	12.40	S.U.
7	25	1175	7.80	13.09	S.U.
8	25	500	7.40	15.79	S.U.
9	25	1725	6.73	11.23	S.U.
10	25	1250	10.23	14.79	S.U.
11	25	775	7.55	14.51	S.U.
12	44	475	9.81	21.09	S.U.
13	44	400	9.45	20.67	S.U.
14	44	450	10.34	21.84	S.U.
15	44	450	10.45	21.83	S.U.
16	44	450	9.70	21.89	S.U.
17	56	400	12.91	22.22	F
18	56	300	12.43	23.65	F
19	56	525	12.27	20.48	F
20	56	450	13.38	21.80	F

Soil sampling took place in May 2017 and 2018. At each plot of 20 × 20 m^2^, soil from forest land and adjacent grassland were sampled. For each the forest land, a survey was conducted to locate an average tree in the stand and a soil profile with a length, width and depth of 1 m each was excavated. The distance of the soil profile from the average tree was approximately 1 m, then, the soil was divided into 6 layers at fixed depths (0–10, 10–20, 20–40, 40–60, 60–80, 80–100 cm). To determine soil properties (moisture content, BD, TN, TP, TK, soil texture, pH), each layer was sampled natural soils with a cutting ring with an inner diameter of 70 mm and a height of 52 mm, 3 repetitions. For each the control grassland, the soil profile was dug at the centre of each plot and the same sampling method as forest land was performed. In addition, 5 sampling points were randomly selected in each plot (forest land and grassland), and each sampling point was stratified-sampled at fixed depths using a soil drill with a length of 1 m. To reduce the number of samples, 6 samples from the same layer were mixed into one sample. All samples were taken back to the laboratory to determine the soil property values.

### Soil preparation

The sealed cutting ring and natural soils were weighed and dried at 105 °C until constant weight. After the samples cooled, the dry natural soil and cutting ring were weighed for BD and soil moisture determination^35^. The other soil samples were air-dried for at least one week. The coarser roots and stones (>2 mm) were removed and weighed. Because the stone (>2 mm) contents in the samples were zero, each sample was sieved through a 0.25 mm sieve. After sieving, the samples were analysed for SOC, total nitrogen (TN), total phosphorus (TP), total potassium (TK), and pH.

### Laboratory analyses

The soil aggregates were crushed and sieved using a 2-mm sieve. The soil texture was measured by an HORIBA’s LA-300 Laser Diffraction Particle Size Distribution Analyser (Japan). The sieved soil samples were evenly divided into three parts (approximately 25 g each). Each part was ground in a grinding dish until the soil particles were separated; then, the samples were sieved through 0.5-mm, 0.25-mm, and 0.1-mm sieves and to obtain three soil particle sizes (0.5–0.25 mm, 0.25–0.1 mm, <0.1 mm), which were categorized as mass 1, mass 2, and mass 3. Three replicates were collected for each sample. The sizes of soil particles >0.1 mm were determined by the sieving method, and those <0.1 mm were measured using an LA-300 HORIBA laser particle size analyser. Three analyses were carried out and averaged to determine the particle size distribution. Because there were fewer clays and silts in the soil, the summed percentages of clay (<0.002 mm) and silt (0.002–0.05 mm) were used to represent the fine soil percentage (FS), and the percentage of sand (0.05–0.5 mm) was used to represent the coarse soil percentage (CS) at each site.

The SOC concentration was determined by the potassium dichromate (K_2_Cr_2_O_7_) volumetric-external heating method, the TN content was determined by the semi-micro-Kelvin method, the TP content was determined by the NaOH melt-molybdenum anti-colorimetric method, the TK was determined by the NaOH melt-flame photometric method, the pH was measured in a suspension of 10 g soil and 25 mL 0.01 mol·L^−1^ CaCl_2_ using a glass calomel electrode GK2401. The determination and calculation of SOC, TN, TP, TK and pH were referred to literature^35^.

### Calculations

The change in each factor was estimated as:

1 $$\Delta {X}_{ij}={X}_{i}-{X}_{j}$$

where $\Delta {X}_{ij}$ is the variation of factor *i*, *X*_*i*_ is the measured value of factor *i* in forest land, and *X*_*j*_ is the measured value of factor *i* in adjacent grassland.

The relative change rate of each factor was estimated as:

2 $${P}_{i}=\frac{\Delta {X}_{ij}}{{X}_{j}}$$

where *P*_*i*_ is the relative change rate of factor *i*.

### Statistical analyses

The relative change rate of SOC was used as the dependent variable, and the other influencing factors were used as the independent variables for stepwise regression analysis. The various influencing factors that exhibited collinearity were excluded, and the relative change rate of SOC was obtained with the following regression equation:

3 $${P}_{SOC}={\beta }_{0}+{\beta }_{i}{P}_{i}$$

where *P*_*SOC*_ is the relative change rate of SOC, *β*_0_ is a constant, and *β*_*i*_ is the coefficient estimates of affecting factor *i*.

The SOC was estimated as

4 $$SOC=\frac{{\beta }_{i}SO{C}_{j}}{{X}_{j}}{X}_{i}+SO{C}_{j}(1+{\beta }_{0}-{\beta }_{i})$$

where *SOC*_*j*_ is the measured value of SOC in adjacent grassland.

The positive and negative effects of SOC variation and its influencing factors have the same positive and negative effects of SOC as their corresponding factors, but the coefficient estimates are different.

To detect the effects of soil properties and stand age on SOC changes. Stepwise regression analysis was carried out in which the SOC was taken as dependent variable while other all factors were independent variables. Before the stepwise regression analysis, the linear models was used to test whether the soil properties were interacted. If there were linear correlations between two soil properties, one of them would be selected for analyse, which was done to eliminate the influence of collinearity on the results. Finally, the factor that significantly affected the SOC was found by stepwise regression analysis, and the stepwise regression analysis was done for each factor with significant influence as a dependent variable to identify the main factors affecting these factors, all analyses were performed by SPSS 20.0 developed by IBM.

## Results

### Change of SOC with stand age and soil depth

**Figure 2:**
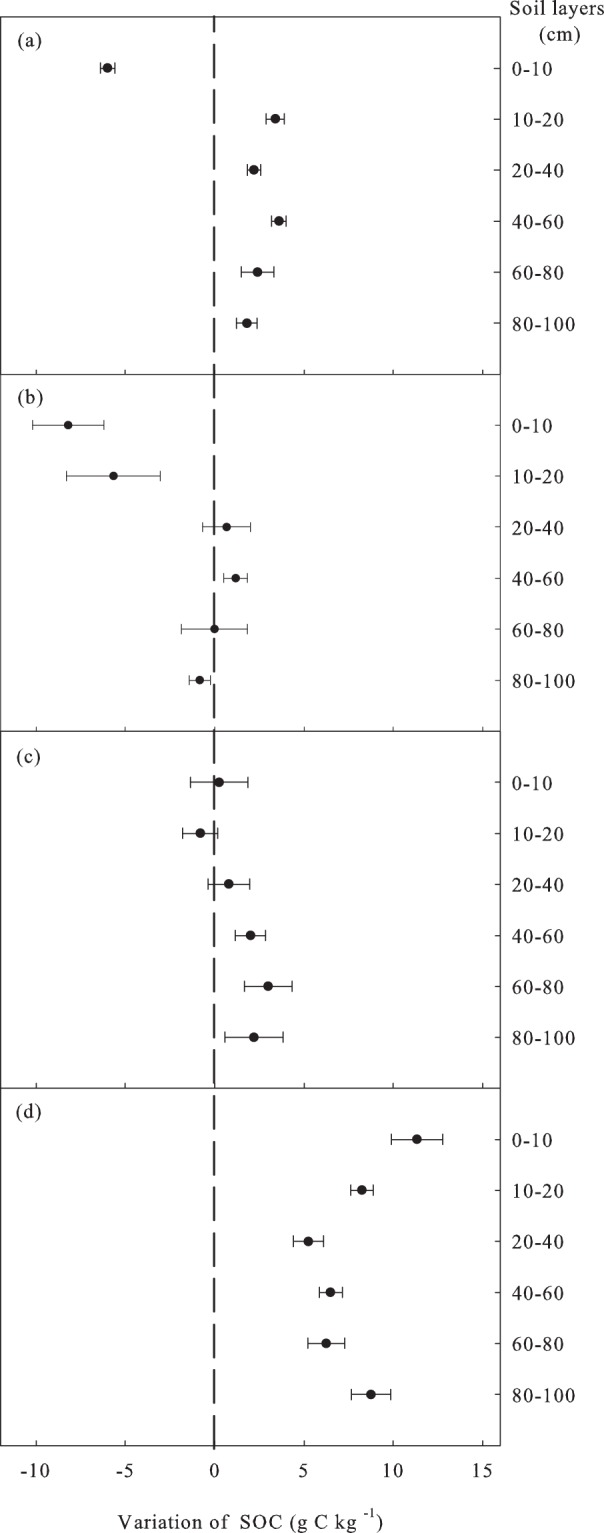
Vertical distribution of SOC variation. (**a**) 13 yrs, (**b**) 25 yrs, (**c**) 44 yrs, (**d**) 56 yrs.

After the conversion of grassland to *Pinus sylvestris* var. *mongolica* forest, stand age was the main driving factor of the SOC in the 0–40 and 80–100 cm layers (Table 2). The analysis of SOC in *Pinus sylvestris* var. *mongolica* forest of different growth stages revealed that at 13 yrs, the amount of SOC was increased in the 10–100 cm layer but significantly decreased in the 0–10 cm layer. The magnitude of SOC change was significantly higher in the 40–60 cm soil layer than that in the 80–100 cm layer (Fig. 2a). At 25 yrs, the decrease of SOC in 0–20 cm layer resulted in lower amount of SOC in 0–100 cm layer than that in control grassland. However, the SOC was increased in the 40–60, 80–100 cm soil layer, and almost unchanged in the 20–40, 60–80 cm layer (Fig. 2b). At 44 yrs, the SOC in the 0–10 cm layer recovered to the initial grassland level and increased slightly, and the level in the 10–20 cm soil layer remained lower than the initial level, while others were increased. The vertical distribution indicated that the magnitude of SOC change was increased with depth (Fig. 2c). By 56 yrs, the magnitude of SOC change in 0–100 cm layer was significantly higher than that in the initial grassland, especially in the 0–10 cm layer.

**Table 2:** SOC stepwise regression analysis (only factors with significant correlations are listed, 0 < VIF < 10 denotes there is no collinearity, where VIF is Variance Inflation Factor).

Soil layer (cm)	Affecting factor	Coefficient estimate	SD	*P*	VIF	*R* ^2^
0–10	Stand age	0.928	0.151	<0.001	4.277	0.925
	TK	−0.502	0.106	<0.001	2.113	
	TP	−0.430	0.100	0.001	1.890	
	TN	−0.392	0.133	0.011	3.329	
	Soil moisture	0.197	0.089	0.044	1.498	
10–20	Stand age	0.830	0.221	0.002	5.535	0.877
	TP	−0.687	0.171	0.001	3.333	
	CS	−0.373	0.120	0.008	1.644	
	TN	−0.295	0.112	0.020	1.431	
20–40	TK	0.735	0.326	0.039	3.073	0.446
	BD	0.714	0.206	0.003	1.229	
40–60	CS	−0.550	0.197	0.012	1.000	0.302
60–80	TN	−0.664	0.253	0.020	1.615	0.447
80–100	Stand age	1.041	0.160	<0.001	1.511	0.729
	CS	−0.344	0.150	0.036	1.332	
	TP	−0.317	0.142	0.040	1.189

**Figure 3:**
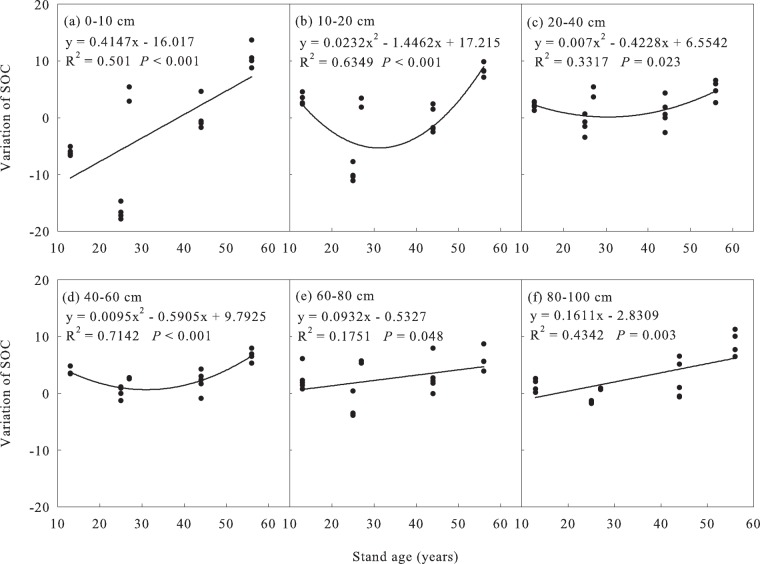
The relationship between the SOC variation and stand age. After the establishment of *Pinus sylvestris* var. *mongolica* forests, the SOC in the 0–100 cm soil layers were significantly correlated with stand age.

On average, the data from the grassland converted to the *Pinus sylvestris* var. *mongolica* forests showed that the SOC contents in the 0–100 cm layers increased by 1.17, 1.29, and 7.58 g·kg^−1^ at 13, 44, and 56 yrs, respectively, and decreased by 0.37 g·kg^−1^ at 25 yrs. At the same time, the data analysis showed that the SOC changes in the surface and deep soil of the forest land were inconsistent, and the SOC changed faster in the topsoil (Fig. 3a), while that in the deep soil changed relatively slowly (Fig. 3c–f). soil organic C content in the <10 cm layers generally decreased by 0.047% per year relative to the initial SOC content during 13 years of afforestation, followed by a increase (up to 0.087% per year) in the rate decline from 13 to 25 years, eventually recovered to the initial level in 44-year-old forests. During the 12 yrs from 44–56 yrs, SOC was in a rapid accumulation phase and increased by 0.089% per year in the 0–10 cm layer. SOC of 10–20 cm layer was increased before 13 yrs, but decreased from 25 yrs, still slightly decreased at 44 yrs, and significantly increased at 56 yrs. The SOC was increased from 13 to 56 yrs and did not reach equilibrium at soil depths >30 cm. Surprisingly, compared with mature forests, SOC changes in all soil layers increased significantly in the over-mature forests. Starting from the middle-aged forests, the SOC increased with stand age, and the SOC in 0–10 cm layer increased faster than that in deeper layers (Fig. 2). Stepwise regression analysis showed that the magnitude of SOC was a significant positive correlation with stand age in the 0–10, 10-20, and 80-100 cm soil layers (*P* < 0.001, 0.002, <0.001, respectively). Based on the estimated value of coefficients, stand age played the most important role in SOC increase (Table 2).

### The effects of soil properties on SOC

Soil moisture was positively correlated with the SOC in only the 0–10 cm soil layer (*P* = 0.044), it was showed that rainfall had a significant effect on SOC content in 0–10 cm soil layer, and SOC increased with the increase of rainfall, in the other soil layers, soil moisture was not correlated with the SOC. There was no significant correlation between SOC and BD in the other soil layer except for 20–40 cm soil layer (*P* = 0.003), The content of SOC in this layer (20–40 cm layer) increases with the increase of BD. The effects of soil texture (CS) were mainly found in 10–20, 40–60 and 80–100 cm soil layers. The CS was significantly negatively correlated with the SOC (*P* = 0.008, 0.012, 0.036, respectively). The coarser the soil texture, the more the SOC increased with the reduction in CS after conversion to the *Pinus sylvestris* var. *mongolica* plantation. Therefore, the sandy soil with high content of coarse grains is very helpful to improve SOC reserves. pH value was not correlated with the SOC in any soil layers.

The effects of soil nutrient on the SOC were also different. SOC was significantly negatively correlated with TK, TP, TN in the 0–10 cm soil layer, significantly negatively correlated with TP in 10–20, 80–100 cm soil layer, and significantly negatively correlated with TN in 10–20, 60–80 cm soil layer. However, TK was significantly positively correlated with SOC in the 20–40 cm soil layer.

## Discussion

### SOC change with soil depth and stand age

Following the afforestation in grassland, soil physicochemical properties differed with soil depth^36^, and soil depth significantly affected SOC^37^. Our results were similar to those observed for surface (0–20 cm) soil. The SOC content of the surface soil decreased initially, and then increased continuously until it returned to the initial state after afforestation at 44 yrs (Fig. 2a–c), while the SOC in deep layer (below 20 cm) has been increasing since afforestation. Similar to the present study^29,34,38^, remarkable changes in SOC at different soil depths due to afforestation were previously demonstrated for various soil properties. However, most studies had shown that the SOC in the initial stage was decreased after afforestation^1,33,39^. The age at which SOC decreased to its lowest value was consistent with Hu *et al*.^31^, but the age at which SOC recovered to initial levels was inconsistent with the findings of Hu *et al*.^31^. Our results were more reliable than those of the previous study because our plots included the whole growth stage of *Pinus sylvestris* var. *mongolica* forests.

After grassland afforestation, within the soil 10 cm from the surface during the first 13 yrs of planting, there was little above-ground input of C due to the small forest biomass and low rate of litter fall^36^. In addition, at this stage, the mass of fine roots (<1 mm) under various forests was only one-fifth lower than that under adjacent pasture sites^40^. Tree roots were sources of organic matter that were less important than grass roots because much of the tree root system lives for many years. The annual turnover of organic matter from dying tree roots was, therefore, smaller than that from grass roots^33^. However, SOC from residues of the preceding pasture phase would continue to decompose during this time. Therefore, the initial decrease in soil C was due to the loss of C through decomposition, which outweighs the gains in C from litter production^40–42^. Soil disturbances involved in the establishment of plantations could result in decomposition of SOM and carbon losses occurring at different rates in different parts of the soil profile. For example, the initial decrease in soil C following forest establishment it was often attributed to site preparation^43^. The partial land preparation of removing surface vegetation no bigger than 20 cm in diameter was regarded as a low-level disturbance^39^. This disturbance still increased the surface temperature of the afforestation site and resulted in accelerated decomposition of the residual organic matter during the initial stage of afforestation. On the other hand, because the surface cover was removed after soil preparation, the soil C stocks decreased due to accelerating soil erosion (wind or water erosion)^44–46^. C leaching could play a role in the reduction in soil C stocks in these areas. However, this impact was not evident from the analysis of the data on soil sampling depth. Finally, the growth of woody plants resulted in a decrease in SOC. The decrease in SOC at 25 yrs in the 10–20 cm soil layer in this study may be due to the growth of woody plants, despite the fact that woody plants produced a greater amount of more recalcitrant material^45^.

The afforestation method used in this study did not disturb the deep soil. The SOC change in the deep soil was mainly dominated by the growth of deep-rooted plants such as *Pinus sylvestris* var. *mongolica* after afforestation^9^.

Due to the growth of plants, the root exudates produced by its root system continuously deposited and decomposed during the growth process in 0–20 and 80–100 cm soil layer (Table 2) and provided a consistent source of top and deep SOC^3^. In addition, accompanied by rainfall and soil water movement, soil soluble organic carbon migrated from litter to the surface soil and deep soil^47,48^; although the proportion was small, it contributed to the SOC increase in deep soil. For the whole soil layer within 1 m, the SOC increased in forest of all ages except 25 yrs, in which SOC decreased. Guo *et al*.^33^ reported that grassland afforestation reduced the soil C stock by 10% rather than increasing it, which may have reflected that fact that deep SOC was not considered.

Afforestation in grasslands had a significant positive effect on SOC changes in the studied region. The *Pinus sylvestris* var. *mongolica* forest floors may have enhanced the positive effects of afforestation on SOC (Unfortunately, there were no related data in this paper). The research showed that the stand age was the main driving factors of SOC changes in the 0–100 cm layer (Fig. 3), especially in 0–20 and 80–100 cm soil layers (Table 2), and many soil properties were also directly or indirectly affected by stand age (Table 3), such as W, TP, TN, TK, CS. The regression analysis showed that stand age was the most important factor that affected the TN and TP in the 0–20 cm soil layer, and W decreased with the increase in stand age in the 0–10 cm soil layer (Table 3). Many studies have also shown that stand age was one of the main drivers of SOC change^3,33,34,39,49–53^.

**Table 3:** Stepwise regression analysis of soil influencing factors (only factors with significant correlations are listed, 0 < VIF < 10 denotes there is no collinearity, where VIF is Variance Inflation Factor).

Soil layer (cm)	Soil properties	Affecting factor	Coefficient estimate	SD	*P*	VIF	*R* ^2^
0–10	TK	SOC	−0.589	0.143	0.001	1.668	0.805
		CS	0.402	0.139	0.011	1.590	
		W	0.312	0.114	0.015	1.070	
	TP	Stand age	1.512	0.243	<0.001	3.850	0.769
		SOC	−1.245	0.223	<0.001	3.230	
		TN	−0.582	0.216	0.017	3.031	
		TK	−0.472	0.203	0.035	2.675	
	TN	Stand age	1.250	0.271	<0.001	5.636	0.805
		SOC	−0.620	0.265	0.033	5.393	
		TP	−0.517	0.188	0.015	2.717	
		CS	−0.408	0.149	0.015	1.715	
	W	SOC	1.133	0.328	0.004	3.230	0.500
		TK	0.893	0.299	0.009	2.675	
		Stand age	−0.800	0.358	0.041	3.850	
10–20	TP	Stand age	0.985	0.122	<0.001	1.537	0.845
		SOC	−0.706	0.123	<0.001	1.565	
		CS	−0.395	0.103	0.001	1.090	
	CS	TP	−0.929	0.182	<0.001	1.594	0.667
		W	−0.804	0.184	<0.001	1.628	
		SOC	−0.409	0.147	0.013	1.042	
	TN	Stand age	0.636	0.237	0.016	1.537	0.417
20–40	TK	Stand age	−1.187	0.125	<0.001	1.927	0.878
		W	−0.534	0.136	0.001	2.275	
		SOC	0.237	0.108	0.045	1.436	
	BD	TP	−0.733	0.142	<0.001	1.233	0.740
		TN	−0.588	0.132	<0.001	1.063	
		TK	−0.561	0.146	0.001	1.302	
40–60	CS	W	−0.384	0.189	0.046	1.521	0.623
60–80	TN	TK	0.811	0.220	0.002	1.606	0.550
		TP	0.696	0.245	0.012	1.998	
		SOC	−0.393	0.182	0.048	1.109	
80–100	CS	TK	0.539	0.139	0.001	1.214	0.746
		Stand age	0.467	0.158	0.009	1.575	
		W	−0.404	0.151	0.017	1.443	
	TP	pH	0.570	0.194	0.009	1.000	0.325

The SOC increased more rapidly with stand age increased, indicating that grassland afforestation could significantly increase SOC. Zhao *et al*.^21^ showed that the total carbon density of the soil layer in sparse elm forests and poplar plantations in the Hunshandake Sandy Land increased with stand age, and the results of global forest and stand age studies also showed that C increased with stand age^54^. However, the SOC changes with time were limited after afforestation in Northern Europe^34^. Walter *et al*.^19^ studied 18 woodlands at ages between 8 and 35 yrs in Central Europe. Rowe *et al*.^20^ studied 12 poplar or willow plots for 4–23 yrs in the UK. Both studies found no significant change in the SOC stocks with stand age. Similarly, Mao *et al*.^18^ did not find significant changes in SOC in a time series study of poplar plantations. This discrepancy may be related to the initially lower SOC content. At initial stages, *Pinus sylvestris* var. *mongolica* plantations had little impact on the 0–20 cm soil. Site preparation and the legacy of the former grasslands (e.g., soil microbial activity, soil physical properties, availability of soil nutrients) probably played major roles^34^. As forests developed, the input of C from litterfall increased and stabilized many years after afforestation due to canopy closure^55^, whereas SOC losses were generally observed following the afforestation in grasslands^33,39,56^. After the establishment of a plantation, a transition phase was often reported to occur^41^. The characteristics of a transition phase were the loss of SOC followed by a recovery phase. Meta-estimates calculated for the periods <30 yrs and >30 yrs since afforestation revealed a shift from initial loss to subsequent gain in SOC^34^.

Although the lengths of the transition and recovery phases were determined by factors such as the soil characteristics, species, and climate, for the 1 m deep soil layers, stand age was the most important driving factor affecting SOC change after the conversion of sandy grassland to *Pinus sylvestris* var. *mongolica* forest. In the 0–10 cm soil layer, the initial stage (<38 yrs) was decreased (Fig. 3a), and the SOC level in the grassland was reached in approximately 38 yrs. The established correlation equation showed that the SOC increased in the 10–20 cm soil layer for the first 16 yrs, decreased to the lowest level by 32 yrs, and then began to increase and reached initial level approximately by 46 yrs (Fig. 3b). The SOC change increased with stand age in the >20 cm soil layers (Fig. 3c–f). compared with grassland, SOC in 56-year-old plantations increased significantly. The magnitude of SOC change in each layer from 0–100 cm had not reached equilibrium, and there kept a large increase potential (Fig. 2). Compared with mature forests, the over-mature forests had stronger carbon sequestration ability (Fig. 2). Therefore, the pattern of conversion of sandy grassland to *Pinus sylvestris* var. *mongolica* was worth promoting in the mid-temperate sandy grasslands of China.

### Effects of soil properties on SOC

Soil properties significantly affected SOC. In general, in arid and semi-arid regions, a decrease in pH was associated with an increase in SOC^57^, as was also found in Chen *et al*.^32^, Giddens *et al*.^58^, Mueller *et al*.^59^ and Fang *et al*.^60^. Low pH values were often associated with low soil microbial activity, and low microbial activity reduced the mineralization of organic matter. Afforestation reduces soil Ca^2+^ and Mg^2+^ levels, resulting in increasing H^+^ levels^37,57,61–63^. However, our findings showed that SOC in any soil layers was not significantly correlated with pH value after planting *Pinus sylvestris* var. *mongolica* forests, as was also found in Liu *et al*.^13^. The viewpoint that high soil pH had a consistently negative effect on SOC storage^32^ was not suitable for sandy soil.

The SOC was only significantly positively correlated with soil moisture in the 0–10 cm layer (*P* = 0.044) in this study (Table 2), Deng *et al*.^37^ found that SOC was significantly positively correlated with soil moisture in China, indicating that soil moisture played an important role in soil C sequestration of forest ecosystems because high soil moisture contributed to a high net primary productivity and a high SOC accumulation^32,37^. Regression analysis also showed that TK (0–10, 20–40 cm soil layers) and CS (10–20, 40–60, 80–100 cm soil layers) which affect SOC change were significantly correlated with soil moisture. However, after planting *Pinus sylvestris* var. *mongolica*, soil moisture decreased with the increase of stand age (Table 3). Low soil moisture would affect SOC accumulation. Therefore, appropriate supplementation of soil moisture after afforestation would increase SOC accumulation.

Previous studies had shown that SOC content had a significant impact on BD^64,65^, BD changes are primarily driven by SOC content and soil texture^49^. After grassland afforestation, the variation of BD in 0–10 cm soil layer was greater (coefficient of variation was 78%). The BD variation in 20–60 cm soil layers decreased first and then increased with the increase of stand age of *Pinus sylvestris* var. *mongolica*, and showed a decreasing trend in 60–100 cm soil layers^66^. We found that BD was only positively correlated with SOC in 20–40 cm soil layer, and our results indicated that changes in BD could also be attributed to soil nutrient changes (Table 3).

After afforestation, the soil of *Pinus sylvestris* var. *mongolica* plantation is still dominated by sand grains (about 95%). The content of CS in different soil layers tends to decrease, with a significant change in 0–10 cm layer. With regard to soil textural class, the negative impact of afforestation on SOC was found in the 10–20, 40–60 and 80–100 cm layers; that was, the SOC decreased with the increase in CS in these three soil layers. This result meant that the SOC increased with the increase in FS. Jobbágy *et al*.^1^ showed that the clay content determines the SOC content of deep soil. The general pattern that had been previously observed was high SOC accumulation in FS^39,53^, and our study observed this phenomenon in the 10–20, 40–60 and 80–100 cm soil layers. However, in northern Europe, the CS was the most prone to SOC increases following afforestation, whereas FS negatively affected SOC sequestration^34^. There effects of soil texture on SOC stocks with land use change continue to be debated, as other studies had not detected any effect of texture^67,68^.

The content of TN and TP were lower in 13 yrs forest land than in control grassland, and then gradually accumulated with the increase of stand age, and they were restored to grassland level in 44 yrs and 25 yrs, respectively. While the content of TK restored to grassland level in the 25 yrs, and then decreased with the increase of stand age. The application of fertilizer was a common practice during plantation establishment, and many studies had found that it increased C accumulation on plantations by increasing both above- and below-ground growth and litter production^39,69,70^. Soil phosphorus was a major nutrient that controlled plant growth and development in forest ecosystems; it had been shown that soil phosphorus decreased concurrently with increasing SOC after afforestation (e.g., with conifers) of former grassland^71^ and that SOC was significantly and positively correlated with soil TP^37^. The results of our study at the surface were inconsistent with this result (Table 2). Luo *et al*.^72^ reported that N dynamics were a key variable that regulated long-term terrestrial C sequestration. Furthermore, C-N interactions were important when determining whether C sinks in land ecosystems could be sustainable over long periods^73,74^. It was likely that N became progressively more limiting as C accumulated in an ecosystem with elevated carbon dioxide (CO_2_) if the amount of N in the ecosystem did not change^72^. If additional C inputs stimulated capital gains of N by biological fixation and atmospheric deposition, increasing N uptake of available soil N or decreasing N loss, then the progressive development of N limitation would not occur^37,73^. Accordingly, there was a significant correlation between SOC storage and TN content in soil^35^. An increase in N could promote the fixation of soil C^75^. This phenomenon had been observed during the early cultivation of artificial pine forests^76^. Due to the influences of afforestation species and growth phase, organic carbon and total N content sometimes showed a significant negative correlation^23^. In this study, the analysis revealed that the negative correlation between SOC and TN in *Pinus sylvestris* var. *mongolica* forests was significant in the 0–10, 10–20, 60–80 cm soil layer, the other soil layers were not correlated, supporting the view that the C-N relationship was complex^52^. In most cases, the N, P, and K contents were significantly positively correlated with SOC^77^. According to this relationship, some scholars had attempted to determine the change in SOC stock by evaluating soil fertility, including N, P, and K contents, after land use changes^77^. However, we found that there was a significant negative correlation between TN, TP, TK and SOC in 0–10 cm soil layers after afforestation (Table 2), indicating that afforestation caused an increase in SOC while the TN, TP, TK content in the soil gradually decreased. TN, TP and TK returned to soil by litter decomposition could not meet the growth needs of trees. Zhao *et al*.^21^ found that the P returned to the soil by litter was insufficient to maintain forest demand in the area, too. The results indicated that using soil fertility changes to infer SOC changes in sandy land could be arguable. In the future, the accumulation mechanism of soil organic carbon in over-mature stage of *Pinus sylvestris* var. *mongolica* forest will be studied.

## Conclusion

After establishment of *Pinus sylvestris* var. *mongolica* plantation on sandy grassland, the SOC accumulation increased with the age of *Pinus sylvestris* var. *mongolica* plantation during the whole growth process. The SOC of near the soil surface underwent the stages of decline, recovery and increase during the growth of the stand. In the process of SOC change in the topsoil layer, the SOC was negatively correlated with TN, TP, TK and CS, and positively correlated with soil moisture. Therefore, the establishment of *Pinus sylvestris* var. *mongolica* plantation on sandy grassland increased SOC content, especially, the over-mature forest had a significant role in promoting the increase of SOC. Clear cutting of *Pinus sylvestris* var. *mongolica* over-mature stand would lead to a significant decline in SOC. We should pay enough attention to the management of over-mature forest.

## Supplementary information

DATA
